# Automated dissection of permanent effects of hippocampal or prefrontal lesions on performance at spatial, working memory and circadian timing tasks of C57BL/6 mice in IntelliCage

**DOI:** 10.1016/j.bbr.2017.08.048

**Published:** 2018-10-15

**Authors:** Vootele Voikar, Sven Krackow, Hans-Peter Lipp, Anton Rau, Giovanni Colacicco, David P. Wolfer

**Affiliations:** a Institute of Anatomy, University of Zürich, Switzerland; b Neuroscience Center, Helsinki Institute of Life Science, University of Helsinki, Finland; c XBehavior GmbH, Bänk, Dägerlen, Switzerland; d Institute of Evolutionary Medicine, University of Zürich, Switzerland; e School of Laboratory Medicine and Medical Sciences, University of Kwazulu-Natal, South Africa; f Chair of Entrepreneurial Risks, Department of Management, Technology, and Economics, ETH Zürich, Zürich, Switzerland; g Institute of Human Movement Sciences and Sport, Department of Health Sciences and Technology, ETH Zürich, Zürich, Switzerland

**Keywords:** Hippocampus, Prefrontal cortex, Spatial memory, Working memory, Circadian, Behavior, IntelliCage, Home cage

## Abstract

To evaluate permanent effects of hippocampal and prefrontal cortex lesion on spatial tasks, lesioned and sham-operated female C57BL/6 mice were exposed to a series of conditioning schemes in IntelliCages housing 8–10 transponder-tagged mice from each treatment group. Sequential testing started at 51–172 days after bilateral lesions and lasted for 154 and 218 days in two batches of mice, respectively. Spontaneous undisturbed behavioral patterns clearly separated the three groups, hippocampals being characterized by more erratic hyperactivity, and strongly impaired circadian synchronization ability. Hippocampal lesions led to deficits in spatial passive avoidance, as well as in spatial reference and working memory tasks. Impairment was minimal in rewarded preference/reversal schemes, but prominent if behavioral responses required precise circadian timing or included punishment of wrong spatial choices. No differences between sham-operated and prefrontally lesioned subjects in conditioning success were discernible. These results corroborate the view that hippocampal dysfunction spares simple spatial learning tasks but impairs the ability to cope with conflicting task-inherent spatial, temporal or emotional cues. Methodologically, the results show that automated testing and data analysis of socially kept mice is a powerful, efficient and animal-friendly tool for dissecting complex features and behavioral profiles of hippocampal dysfunction characterizing many transgenic or pharmacological mouse models.

## 1 Introduction

Behavioral correlates of hippocampal malfunction induced by genetic or experimental lesions have become a gold standard in documenting cognitive changes in laboratory rodents. Deficits in spatial learning tasks such as the water maze are taken as a proxy of hippocampal malfunction, and considered to reflect impaired or missing spatial reference memory as evidenced by prolonged escape latencies and reduced searching over the position of removed escape platforms (probe trials). Likewise, the hippocampus is thought to be critical for short-term spatial working memory as observed in radial maze tasks requiring win-shift strategies to visit rewarded locations that may vary according to a first choice and shifting spatial cues. However, the history of testing hippocampal lesion effects has revealed two important findings. For one, lesion experiments have shown that spatial reference memory can recover to some extent [1], [2]. In addition, many rodent studies found changes in anxiety [3], increased behavioral stereotypies [4], increased locomotor activity [5], [6], impaired species-typical behavior [3], [7], [8], diminished sociability [9], [10], [11] and include, unsurprisingly, hormonal or physiological changes as well [12]. Thus, lesions generate a complex hippocampal syndrome whose components and their interaction are still poorly understood. This situation hampers a comprehensive understanding of hippocampal function(s), specifically after prolonged recovery times or transgenic impairment of hippocampal functions as often observed in constitutive knockout mice.

Likewise, lesioning or inactivating the rodent prefrontal cortex faces the same problem, as this structure interacts strongly with the hippocampus [13], being embedded in a larger forebrain-to-midbrain network governing cognitive and executive processes. However, unlike cytotoxic hippocampal lesions in mice [7], technically similar lesions of the mouse prefrontal cortex entailed hardly any cognitive deficits in a variety of tasks but appeared anxiolytic and revealed a deficit in food burrowing [14].

Obviously, dissecting a hippocampal or prefrontal lesion syndrome requires extensive behavioral testing which is mostly beyond the capacities of behavioral laboratories working with traditional apparatus. In order to establish reference behavioral profiles characterizing hippocampal and other brain area malfunction efficiently, we conducted several IntelliCage studies with mice subjected to hippocampal and other brain area lesions (e.g., pre-frontal cortex, striatum) to assess spontaneous behavior [15], and performance in spatial and non-spatial learning tasks including both reward and punishment [2].

IntelliCage by NewBehavior (TSE Systems GmbH, Germany) is a programmable automated behavioral test system designed for high-throughput behavioral analysis of transponder-tagged mice living in social groups in a large standardized home-cage wherein each mouse can visit 4 operant conditioning chambers. It thus permits to combine analysis of spatial behavior with operant conditioning, thereby covering a large cognitive spectrum. To analyze the large data volumes typically produced by such systems we developed novel software (FlowR, available at XBehavior GmbH, Switzerland) for automated statistical data analysis. This enabled us to analyze efficiently the experimental raw data by extracting common spontaneous behavioral patterns as well as conditioning success in various forms of learning paradigms, following both hippocampal and prefrontal lesions in grouped mice

Using female C57BL/6 mice and long postoperative recovery times to identify lasting deficits, we report here that bilateral large hippocampal lesions impaired only marginally the ability to solve simple rewarded spatial preference and reversal tasks. However, lesioned mice showed massive problems when the task required precise circadian timing, putative working memory, or when wrong choices of locations were punished by airpuffs. Lesions of the medial prefrontal cortex did not entail deficits in any cognitive task applied, notwithstanding apparent separation of spontaneous activity phenotypes of all three groups.

## 2 Material & methods

All behavioral tests were carried out at the Institute of Anatomy, University of Zürich, under standard laboratory conditions with inverted light/dark cycle. Adult female C57BL/6J mice were lesioned in the hippocampal region (HIPP), the prefrontal cortex (PFC), or sham-operated (CTR), and subsequently exposed to a series of fully automated conditioning schemes in IntelliCage (NewBehavior/TSE-Systems GmbH, Bad Homburg, Germany). Two batches of mice (Les1, Les2) were tested in a sequence of protocols using 3 and 4 cages, respectively, each containing mice from each of the three lesion groups. All experiments were carried out in accordance with the guidelines of the European Communities Council Directive of 24 November 1986 (86/609/EEC) and were approved by the veterinary office of the Canton of Zürich.

### 2.1 Animals and lesions

Sixty-five female C57BL/6JRccHsd mice at 70 days of age were obtained from Harlan Laboratories (Füllinsdorf, Switzerland). Animals were kept in groups of 8–10 mice under standard laboratory conditions (temperature 21 ± 1 °C, humidity 50 ± 5%, inverse 12/12 h light/dark cycle) in an animal facility at the institute. The animals were housed in groups of 8–11 mice in standard Type III cages (Tecniplast, Buguggiate, Italy) with water and food (Kliba Nafag 3430; Provimi Kliba AG, Kaiseraugst, Switzerland) ad lib. For the first batch, Les1, 30 mice were randomly assigned to be lesioned either in prefrontal cortex (PFC, n = 10), or hippocampal regions (HIPP, n = 11), or sham-operated (CTR, n = 9). At 192 days of age, 51–116 days after surgical intervention, Les1 mice were transferred to the IntelliCages. For the second batch, Les2, 35 mice were lesioned (PFC, n = 12; HIPP, n = 10; CTR, n = 13) and transferred to IntelliCages at 250 days of age, 150–172 days after lesioning.

### 2.2 Surgical procedure and brain preparation

Preoperatively, each subject was injected (i.p.) with the anesthetic and analgesic Avertin (tribromethanol, 20 ml/kg), the anticonvulsive chlordiazepoxide hydrochloride (10 mg/kg in 10 ml saline) and the anti-inflammatory Rimadyl (carprofen, Pfizer Animal Health, 0.1 ml/kg in 10 ml saline). Following the premedication, mice were placed in a stereotaxic frame (David Kopf Instruments, Tujuga, California). The incisor bar was set to −1 mm to ensure a level head. After incision of the scalp the skull was exposed. The coordinates for the lesion sites were calculated using a reference brain atlas [16]. The interaural line was used as reference for all coordinates, except for the vertical ones.

After verification of the lesion site coordinates using three pilot surgeries, we chose four injection sites for the bilateral hippocampus lesion and three injection sites for the bilateral lesion of the prefrontal cortex on each side of the sagittal suture (Table 1). By means of a 5 μl syringe equipped with a 34 gauge stainless steel needle (Hamilton, Bonaduz, Switzerland) an N-methyl-d-aspartic acid solution in phosphate buffered saline (NMDA, 10 mg/kg)) was injected on each site for the respective lesions (Table 1). After subcutaneous injection of the transponders (Datamars SA, Bedano, Switzerland) through the scalp incision, the scalp was sutured. Control mice were sham operated, after application of the same pre-operative drugs as the lesioned groups. The sham operation consisted in a scalp incision, subcutaneous injection of the transponders and suture of the skin.

**Table 1:** Coordinates and injected volumes (μl) for the brain lesions as depicted in Fig. 2. AP: distance (mm) from interaural line; L: from sagittal suture; V: from skull surface at bregma level. Overjet (mm) refers to the lowering of the needle past the V coordinate. The latter procedure ensures a diffusion of the cytotoxic agent into ventral parts of the targeted structure.

Site	AP(+)	L	V(−)	Overjet	Volume
HPP1	2.1	1.2	1.9	0	0.1
HPP2	1.5	1.7	1.9	0	0.15
HPP3	1.0	2.2	2.0	0.3	0.1
HPP4	0.7	2.8	4.0	0.2	0.2
PFC1	6.3	0.5	2.0	0.5	0.1
PFC2	5.5	0.5	2.0	0.5	0.1
PFC3	4.7	0.5	1.5	0	0.1

All operated mice (lesion and sham) were placed under a heating lamp (temperature controlled at 30 °C ± 2 °C) for recovery. Postoperatively all mice were single housed and regularly screened for seizures and later for locomotor deficits. Conscience was regained after three to four hours post-op. During four days following the surgery all mice had the possibility to drink water with an addition of 10% glucose to sustain recovery.

**Fig. 1:**
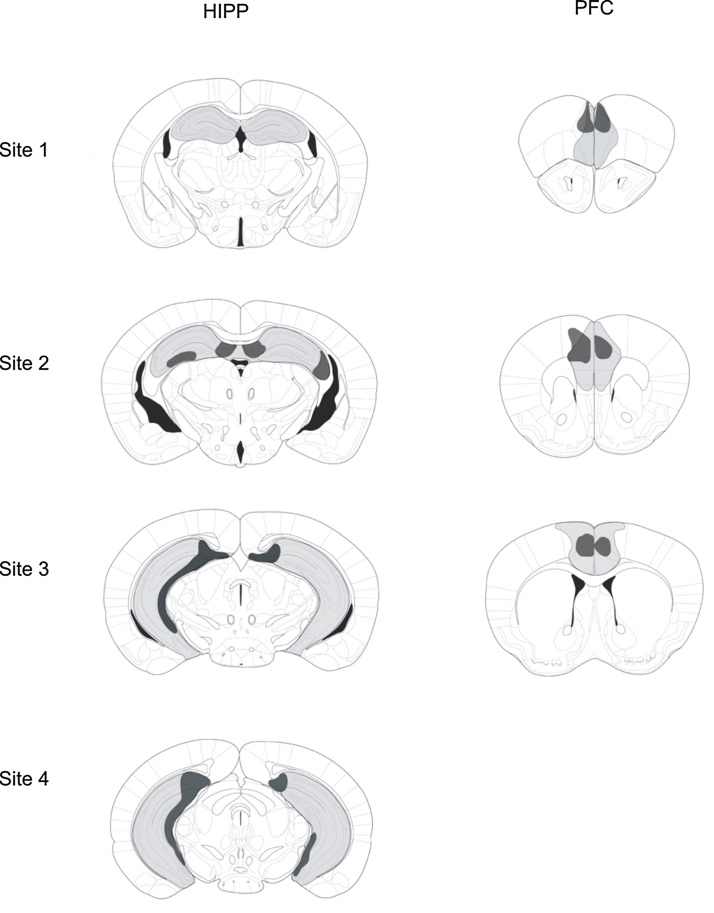
Maximum and minimum lesion extent of bilateral hippocampus lesions (HIPP) and of bilateral prefrontal cortex lesions (PFC), for the respective lesion sites (HIPP: 1–4, PFC: 1–3). Light grey represents the largest lesion extent, dark grey represents the smallest lesion extent observed in a small number of animals.

Perfusion and histology was conducted after experiments, when mice were injected (i.p.) with pentobarbital (100 mg/kg) and transcardially perfused with paraformaldehyde (PFA) 4%, their brains extracted and postfixed for four hours in PFA 4%. The brains were cryoprotected with 30% sucrose to avoid freezing artefacts until sinking of the brains. Freezing of the brains was obtained by CO_2_ application. A sliding microtome (Jung CM 300) was used for cutting (10 series, 40 μm coronar slices). Every 10th series was stained with toluidin-blue. Subsequent lesion scoring was done using a light microscope (Leitz, dialux 20 EB). In the hippocampus as well as in prefrontal cortex most mice had large and complete lesions. A few however, had smaller lesions, likely due to clogging of the injection needle. For ease of reference, Fig. 1 provides the minimum and maximum lesion sizes for the sites (see Table 1).

### 2.3 IntelliCage testing

**Fig. 2:**
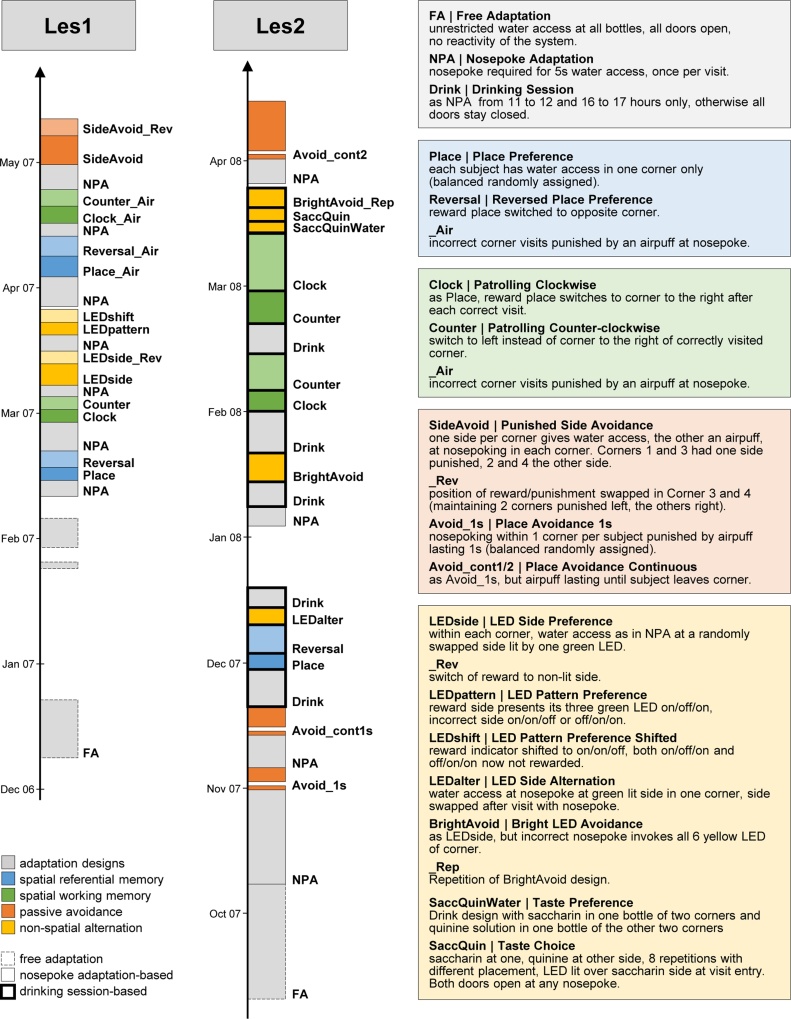
Experimental sequence for the two batches of mice, Les1 and Les2 (left) and conditioning designs applied during the experimental runs (right). Note that except during free adaptation reward refers to a door opening for 5 s allowing drink access at intended side to fluid provided, which could be achieved once per visit only. With Les1 shaping took place continuously, in Les2 during drinking sessions after *Drink* runs, and continuously after *NPA* runs. Counter was interrupted by a Drink run in Les2 due to insufficient water intake of some subjects. In all 3 Avoid repetitions, a 1-day gap followed the punished trial before start of probe trial (without airpuff).

After recovering from surgery, mice were kept in original groups of 8–10 before they were transferred into IntelliCages. Mice were re-grouped so that cage groups consisted of 2–4 females of each of the three lesion groups (CTR, PFC, HIPP), leaving 8–10 mice per cage. Animals were then exposed to a series of conditioning schemes as exposed in Fig. 2.

The IntelliCage apparatus consists of a polycarbonate cage for rats (20.5 cm high x 58 cm x 40 cm at top, 55 cm x 37.5 cm at bottom, Model 2000, Tecniplast, Buguggiate, Italy), containing a conditioning chamber in each corner (view at http://www.tse-systems.com/products/behavior/intellicage/index.htm). Each chamber allows access to 2 water bottles for drinking at left and right sides, by means of a closable round opening, termed “door”. Pokes at these openings are registered by a light beam sensor and termed “nosepokes”. Mice can enter the corners through a tube containing a transponder reader antenna, and a temperature sensor confirms their presence. Antenna readings and presence signals determine whether a subject has entered a corner, termed “visit”. Lickometers indicate licking at bottle caps. Hence, the system can sense identity of individuals visiting a corner, and register nosepoking and licking, all with exact datetimes of start and end of all events. Using IntelliCage Plus controlling software (NewBehavior AG, Zürich), the system can react to pre-defined events, given its place (corner 1–4) and side (left or right) and subject identity, as well as datetime, with switching on/off 3 LEDs of 4 colors at each door, opening/closing valves for applying air puffs (using external pressured air), or opening/closing doors (levers) to allow access to bottle nipples. This allows for a plethora of conditioning schemes based on reward by door opening, punishment by air puff application, and conditional LED stimulation.

Each experimental run is completely controlled by one single experimental file that contains the conditioning scheme designed in a graphical interface that is easily set up by the user. All input and output activities of the system as well as conveniently tabulated events and behavioral acts of mice are stored in text delimited data files, for easy access in dated folders that also contain the experimental files. This allows for unequivocal reconstruction of conditioning schemes and full access to time-stamped spontaneous activity and responses of individual mice, i.e., complete and unequivocal evaluation of animal activity and conditioning success without requirement of any external information.

### 2.4 Behavioral test paradigms

To adapt mice to the system and conditioning schemes, *Free Adaptation*, *Nosepoke Adaptation*, and *Drinking Session* experimental designs were run, as described in Fig. 2. The sequences of conditioning paradigms were interspersed with the appropriate adaptation designs. For comparison of undisturbed spontaneous behavior measures, as given in Table 2, data were taken from days 4–14 of the initial *Free Adaptation* phases in order to avoid any biases due to disparate effects of the initial accommodation phase or due to habituation effects, as Les2 animals had been observed for a longer uninterrupted period initially than Les1 mice (see Fig. 2).

**Table 2:** Behavioral measures extracted per subject during *Free Adaptation*, with ANOVA test statistics and adjustment of significance for false discovery rate. Note that Repetitive and Regularity denote to independent aspects of visiting sequences.^a^^,^^b^

	Behavior	CTR		HIPP		PFC				
		Mean	SE	Mean	SE	Mean	SE	F_(2,33)_	p	p adj.
NPvisits	visits with nosepoke without licks/h	1.65	0.18	2.01	0.21	1.44	0.16	5.43	0.01	0.03
Svisits	visits without licks and nosepokes/h	1.01	0.12	2.34	0.34	0.99	0.14	7.99	0.001	0.01
Lvisits	visits with licks/h	1.59	0.05	1.67	0.10	1.62	0.08	0.03	0.97	0.97
NPVdur	median duration of visits with nosepoke without lick (s)	6.04	0.20	4.14	0.28	6.09	0.29	11.37	0.001	0.001
Nosepokes	mean number of nosepokes during visits with nosepoke without lick	2.88	0.13	2.35	0.14	2.56	0.10	2.82	0.07	0.12
NPduration	median duration of nosepoke during NPvisit (s)	0.85	0.05	0.61	0.05	0.80	0.04	3.53	0.04	0.08
Licks	median number of licks per Lvisit	9.19	0.91	8.68	1.71	8.86	1.07	1.85	0.17	0.21
Lduration	median duration of licking during Lvisit (s)	6.35	0.44	7.44	0.75	6.78	0.54	2.44	0.10	0.14
Nocturnal	log(visit frequency during dark phase/visit frequency during light phase).	1.37	0.44	2.16	0.48	2.41	0.39	1.15	0.33	0.36
Repetitive^a^	log(sum of observed re-entries/sum of expected re-entries)	−0.41	0.05	−0.15	0.05	−0.47	0.05	13.37	0.001	0.001
Regularity^b^	sqrt(sum of squared diviations for non-same corner transitions/number of non-same corner transitions)	0.19	0.01	0.15	0.01	0.18	0.01	3.42	0.04	0.08

a For each subject, the corner transition matrix (frequencies of next corner (1, 2, 3, 4) visited after each corner was left) was constructed. Its diagonal frequencies give the number of cases when a subject re-entered the corner it had last left. The expected frequencies result from the marginal frequencies per corner. For instance, if a subject visits all corners equally frequently, re-entries would occur in 25% of corner transitions if corners were randomly chosen. The log of the quotient of observed/expected re-entries therefore gives a symmetrical measure of deviation from randomness. Values greater than 0 indicate that a subject re-entered corners more often than randomly expected, i.e., exhibits a tendency for repetitive visiting, and vice versa. The higher the value the more repetitive a subject’s visiting behavior is.

b The off-diagonal entries of the corner transition matrix give the frequencies for visits in a corner different from the one last left. The sum of squared residuals, i.e. (observed − expected frequency) ^ 2/expected frequency, of all non-same corner transitions divided by the number of all non-same corner transitions, gives a measure that increases the more regular visit sequences are (the more correlated rows and columns). For instance, a perfectly patrolling subject that enters corners always in sequence 1–2–3–4 would have 0 transitions for 1–3, 2–4 and so on, i.e., the measure would be far higher than in a subject exhibiting all possible transitions. Any pattern in non-same corner visiting sequence would increase this measure, i.e. this statistics would be larger the more regular a subject’s between-corner visiting sequence, but cannot tell anything about the form of the patterning. This measure is inspired by well-known Cramer’s V.

Conditioning designs are described in Fig. 2 and referred to as passive (side or place) avoidance, spatial referential memory, spatial working memory, and non-spatial alternation designs in the Results. *Avoid* and *SideAvoid* designs tested passive avoidance by delivering air puffs either at specific corners of cages, or specific sides within a corner (always randomized to avoid topological or handedness bias). Place avoidance in *Avoid* design was also re-tested after a day of removal of mice from the IntelliCage, in a probe trial in order to evaluate long-term memory effects. Putative spatial reference memory was invoked by reversal tests (*Place* and *Reversal* designs), when reward places were switched to the opposite corner after several days (see Fig. 2). Working memory was sought to be tested using patrolling trials (*Clock* and *Counter* designs) that required subjects to memorize place of last reward in order to find the place of the next one. Putative non-spatial conditioning involved several designs intended to signal reward by LED lights available over each door (*LEDside*, *LEDpattern*, *LEDshift*, *LEDalter*, *BrightAvoid*). Taste choice and preference designs (*SaccQuinWater*, *SaccQuin*) were introduced in order to link a conditional stimulus (*LED light*) to the unconditional preference for saccharin.

### 2.5 Statistical analyses

Data in form of standard IntelliCage zipped archives (TSE Systems GmbH, Bad Homburg, Germany) were read via the automated user interface FlowR (XBehavior GmbH, Bänk, Switzerland). For each shaping scheme analyzed, one data point per subject in each respective treatment group and/or temporal phase was extracted for comparisons. Parameters for general behavioral performance during *Free Adaptation* are described in Table 2. Behavioral measures during free adaptation were visualized using a canonical discriminant analysis and tested for overall significance using a MANOVA statistics, individual measures were compared using linear model ANOVA.

Outcomes for conditioning schemes were either correct or incorrect visit with nosepoke to a particular corner, or correct or incorrect first nosepoke at a particular side within a corner visit. Hence, conditioning success was reflected by correct or incorrect outcome at each visit. Proportions per subject, e.g., of correct responses, were compared with generalized linear (mixed) models using a binomial link function on the logits. For designs measuring individuals repeatedly, subject identity was added as random effect to the within-subject factor (i.e., repeated measure) and between-subject fixed effects.

Learning performance was compared using sequential probability ratio statistics [17]. For this, the cumulative number of correct responses is analyzed over the sequence of trials (visits with nosepokes). The statistics infers the probability of type 1 and 2 errors for accepting that a pre-set preference has been reached or not, respectively, at each visit in the sequence. This can be represented graphically by plotting the cumulative success number against trial number, with linear indicators of the decision levels. We set both type 1 and 2 errors to 5%, and tested for a preference 10% above random expectation. For instance, if visiting one of four corners represented a correct response, random expectation is 25%, preference is accepted at 35% correct visits.

The number of trials (visits with nosepoke) to reach the preset criterion is therefore taken as a measure of learning performance and compared between groups using Cox proportional hazards model. If appropriate, subjects' random effects were accounted for by a frailty penalty function based on a gamma distribution.

Graphics (package “ggplot2”) and analyses were conducted using R [18], run from the FlowR user interface. All linear model analyses are from package “lme4”, proportional hazards from “survival”, canonical discriminant functions from “candisc”, inferences from “car” package. Significance levels of p < 0.05, 0.01, and 0.001 are differentiated.

Note that small subsamples of our data had been analyzed using different methods in papers with different focus: 1. [2] presented Les2 Avoid_cont1/2 and Avoid_1 s (see Fig. 2) analyses together with other data from lesioned mice in a first description of the corner avoidance protocol. Data, probe trial data in particular, were analyzed in a different way and not in the context of other learning tasks. 2. [15] included Les1 and Les2 FA data (see Fig. 2) in a PCA model with a total of 1552 mice. The behavioral profile of hippocampal lesions in FA was compared to other experimental conditions based on component scores, not on measured parameters as in the current submission.

## 3 Results

### 3.1 Behavioral phenotypes differ between groups

#### 3.1.1 Spontaneous behavior during free adaptation

**Fig. 3:**
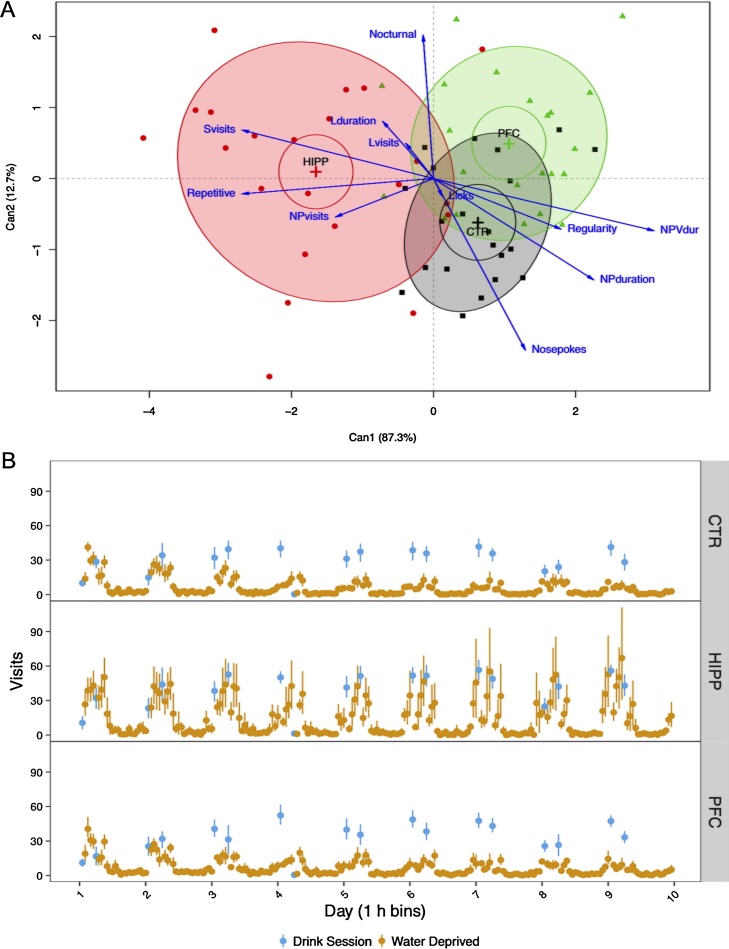
Measures of spontaneous behavior and synchronization of activity with drinking sessions discriminate experimental groups, as determined during *Free Adaptation* in both batches, and first *Drink* run of Les2, respectively (see Fig. 2). A) Canonical discriminant scores of individuals, means and 68% coverage probability ellipses for groups, and correlations of behavioral variables (see Fig. 2) with canonical components. The first canonical component clearly separates HIPP mice from the other groups, while the second canonical component further separates CTR and PFC with some overlap. B) Visits per hour during deprived (light) and drink access (dark) hourly periods (means with bootstrapped 95% CI). The eighth drinking session was unintentionally skipped due to an experimental error. Note that synchronization is strongly hampered in HIPP mice.

During *Free Adaptation* (FA) of Les1, regrouping of subjects caused major gaps in observation and possibly disturbance after the first 2 weeks of observation (see Fig. 2). Also, activity level tended to decrease over the first 1–3 days within several cages (not shown), so that we also excluded the initial 3 days of accommodation, leaving days 4–14, both for Les1 and Les2, for analysis of the spontaneous IntelliCage phenotype. Fig. 3A indicates fairly clear separation of groups by the two components of canonical discriminant analysis (MANOVA Pillai trace = 11.07, df = 2, approx. F_(22,106)_ = 3.08, p < 0.001). The differentiation of groups did not differ between the batches in a two-factor fixed effects model (MANOVA interaction batch:group, Pillai trace = 0.47, df = 2, approx. F_(22,100)_ = 1.41, ns). Spontaneous behavior during nosepoke adaptation (NPA, not shown) led to congruent differentiation between groups, but restriction of nosepoke duration by water access design necessarily restricts behavioral expression (e.g., lick duration, lick visit frequency, NPduration, NPVdur).

The first canonical component clearly separated HIPP from other mice (Fig. 3A; approx. F_(22,104)_ = 3.53, p < 0.001) and correlated positively into HIPP direction most strongly with non-lick visit frequencies (*Npvisit, Svisit*) and repetitiveness (*Repetitive*) while correlating negatively most strongly with nosepoking visit duration (*NPVdur*) and nosepoking duration (*NPduration*) as well as *Regularity* and number of *Nosepokes* per visit. The second canonical component further separated CTR and PFC (Fig. 3A) but proved not to significantly contribute to canonical discrimination (approx. F_(10,53)_ = 1.16, ns). Hence, HIPP mice appeared to visit at higher rate (apart from visiting for licks), with more frequent re-entering of the formerly visited corner than subjects of the other two groups. On the other hand, the other CTR and PFC had longer visits with longer times nosepoking during a visit, and switched place of visiting corners more regularly than HIPP mice.

Overall, HIPP mice visited at higher rates for shorter visits while exhibiting a more erratic spatial pattern of visiting than the other mice.

#### 3.1.2 Synchronisation with drinking session pattern

During first *Drink* run (Les2, Fig. 2) HIPP mice exhibited a stark deviation in temporal synchronization when water access was restricted to two one-hour drinking sessions per day (11 and 16 h, Fig. 3B). CTR and PFC mice perfectly synchronized activity with drinking sessions at least from third day onwards, while HIPP mice kept on increasing activity well before water access was granted. Accordingly, the effect of experimental group on the repeated measure of visit rate over 1 h time periods is highly significant (F_(2,13892)_ = 9.87, p < 0.001).

Furthermore, Fig. 3B also shows that HIPP mice, after second drinking session per day, increased the initially dropped visiting rate again for a third time, as if expecting another drinking period at about the delay applying to their last session.

### 3.2 Passive avoidance deficits with hippocampal lesions

#### 3.2.1 Side avoidance within corner

**Fig. 4:**
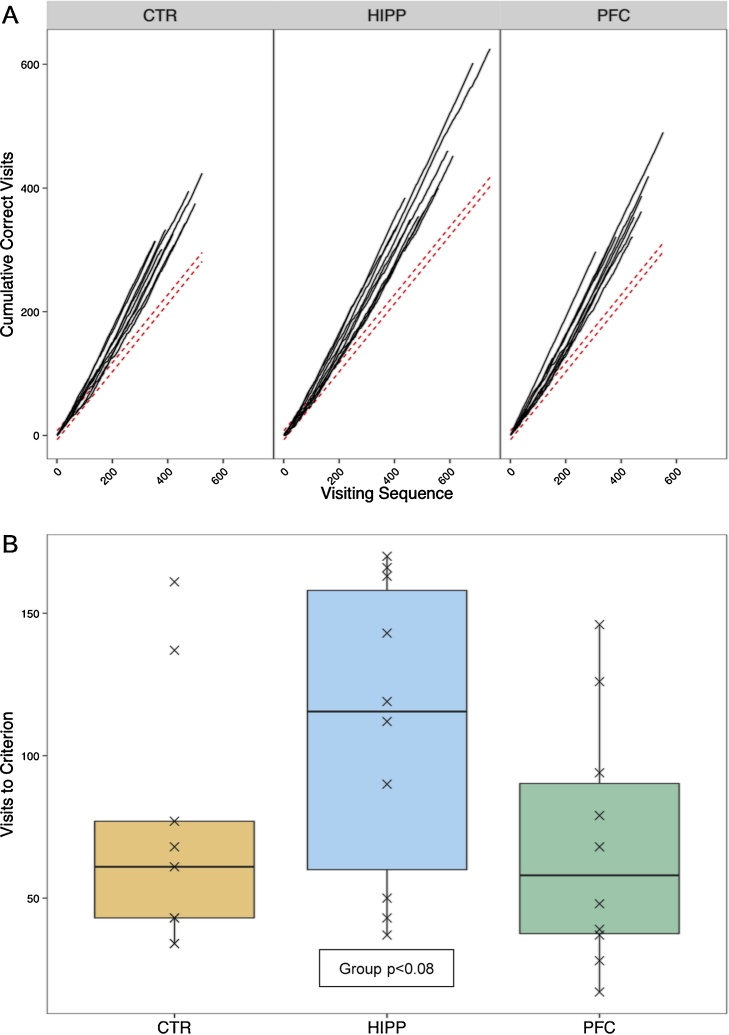
Learning to discriminate the punished from the non-punished side in a corner as evidenced by correctly placing first nosepoke per visit at the rewarded side. This *SideAvoid* task is mastered by all Les1 mice subjected to it (see Fig. 2). A) Learning plots when one side in each corner responded with an airpuff at nosepoke, the other with giving access to water. Learning boundaries indicated by dotted lines. B) Number of visits to reach preference criterion which appears to be slightly but insignificantly increased in HIPP mice (see text). Note that in two corners left sides were punished, in the two others the right side, when left/right patterns differed between animals.

All subjects (of Les1) significantly avoided nosepoking at the punished side of each corner (Fig. 4A) during *SideAvoid* trial (see Fig. 2), with no significant difference in learning speed discernible, although HIPP performed slightly worse (Fig. 4B, Chi^2^ = 5.07, df = 2, p < 0.08). During reversal of punished sides in two corners (see Fig. 2), no effect of group was detected (not shown).

#### 3.2.2 Place avoidance paradigms

**Fig. 5:**
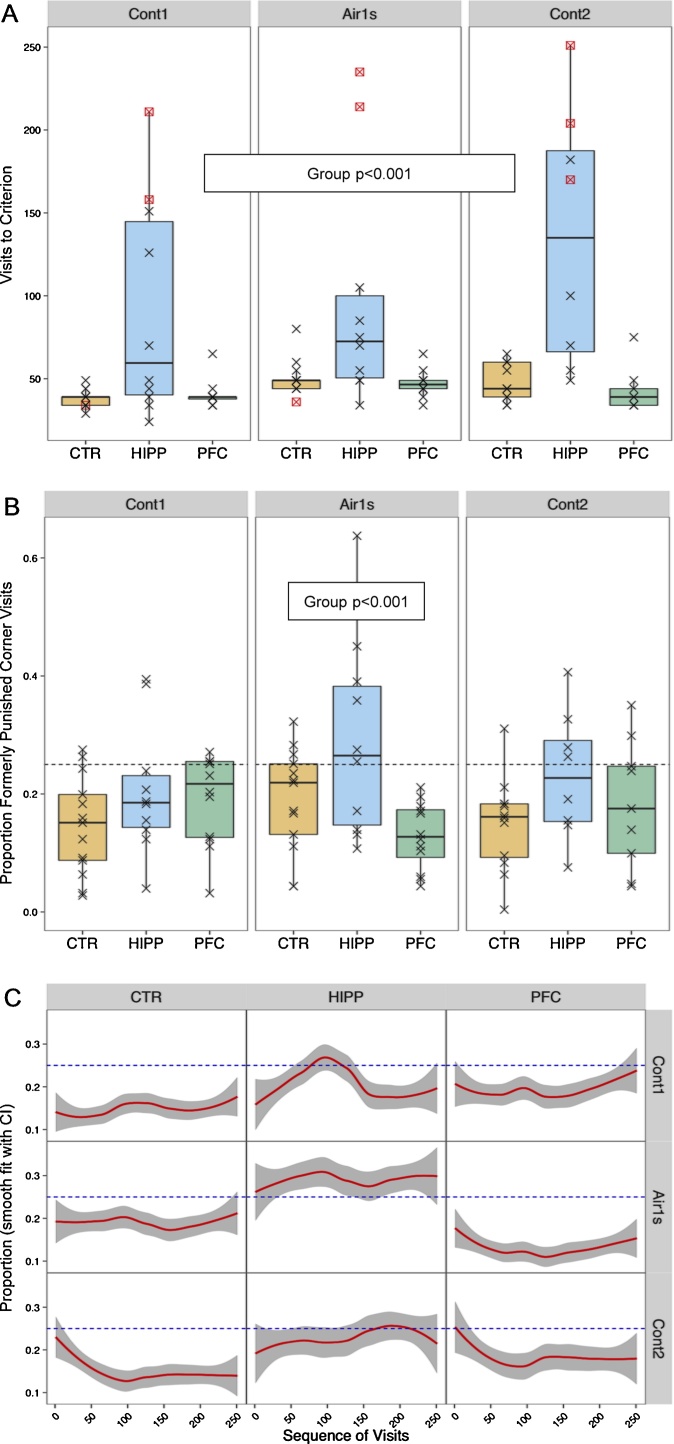
Place avoidance learning is impaired in HIPP mice and probe trial analysis reveals retention problems, in *Avoid* paradigm tested 3 times in Les2 (see Fig. 2). **A**) Visits to criterion when any nosepoke in one corner caused an airpuff, while the three other corners gave access to water upon nosepoking. Framed symbols refer to censored observations (i.e., preference criterion not reached). HIPP mice expressed avoidance more slowly. B) Proportion of first 250 visits with nosepoke to previously punished corner during probe trials after a 24 h period outside IntelliCage (all corners now without punishment). Chance levels at 0.25 is indicated by dotted lines. HIPP mice had visiting rates closer to chance level during probe trials. C) Smoothed lines for visiting rates of subject over the initial 250 visits of the probe trials reveal little evidence of avoidance in HIPP mice (see text for closer examination).

Place avoidance was tested three times with Les2 (see Fig. 2). The *Avoid* designs punished nosepoking in one of the four corners only, either by 1 s air-puffs in the first run, or continuous ones in the two further runs (see Fig. 2). Each time HIPP mice appear to need more visits to reach the preference criterion (Fig. 5A) during the punished day, reflected by a highly significant group effect (Chi^2^ = 44.21, df = 2, p < 0.001). Return to random level during probe trials, i.e., after cessation of any punishment after a day of withdrawal from IntelliCage, did not differ between groups as measured by the number of visits to a criterion of >20% visits to previously punished corner (Chi^2^ = 3.01, df = 2, ns; not shown).

However, a closer look at preference development during probe trials shows that during the first 250 visits with nosepoke, HIPP mice avoided the previously punished corner significantly less than the other mice (Fig. 5B). The difference in proportion of visits to previously punished corner depended significantly on air-puff treatment (Air1s, Cont1, Cont2) in a repeated measures model (Wald Chi^2^ = 123.87, df = 4, p < 0.001) and reached significance only for the Air1 s treatment (F_(2,32)_ = 6.63, p < 0.01; F < 1.7 in other treatments). Note that some subjects, particularly from HIPP group, exhibited a clear preference for the previously punished corner. Please mind that taking the first 250 visits is arbitrary and done for illustrative purposes in the first place. The current conclusion also follows from taking the first 50 or 100 visits (not shown).

Smooth plots show little evidence that avoidance of the last corner with punishment declines over initial visiting (Fig. 5C). On the contrary, in CTR and PFC mice avoidance seems to slightly increase initially. Looking at all visits (not only those with nosepoke) indicates that at the first visits, CTR and PFC mice are close to random 25% visiting at formerly punished corner (Fig. 5C). This observation would be congruent with mice initially having to orientate themselves after re-introduction into their IntelliCages and then sticking to some avoidance of the previously punished corner. Initial disorientation also hampers the non-arbitrary analysis of visits to criterion given above. HIPP mice show little evidence of any avoidance, at all.

### 3.3 Spatial reference memory strongly impaired by hippocampal lesion

**Fig. 6:**
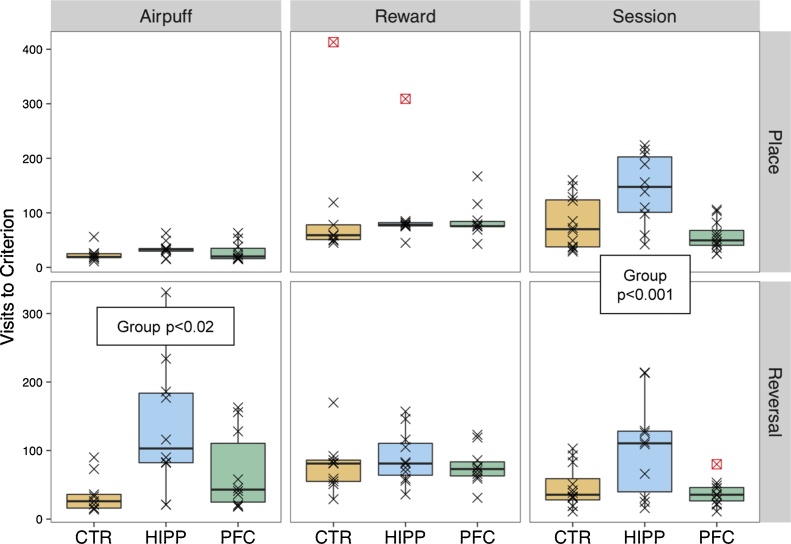
HIPP mice learn unambiguous spatial preference tasks easily but are impaired in *Place*/*Reversal* (see Fig. 2) learning tasks that exhibit aversive reinforcement, or require timing of activity. Visits to criterion are shown for Les1 trials without (Reward panel) and with punishment (Airpuff panel) as well as for the Les2 test under restriction to two 1 h drinking periods per day (Session panel). Framed symbols refer to censored observations (i.e., preference criterion was not reached). Note that place learning without punishment takes longer, presumably because of sufficient partial reinforcement. HIPP mice performed far worse at reversal under airpuff treatment (Airpuff/Reversal panel) and during drinking time-dependent learning (Session panels).

Putative measures of reference memory were tested in three paradigms, each first conditioning for preferential visiting of a rewarded corner, and then switching the design in order to reward the opposite corner (reversal). *Reward* and *Airpuff* paradigms were applied to the first batch of mice, Les1, *Session* paradigm to the second batch Les2 (see Fig. 2). An overall statistical test of the effects exposed in Fig. 6 reported a highly significant differentiation of the group effect between treatments (Place/Reversal) and experiments (Reward, Airpuff, Session; Chi^2^ = 89.76, df = 4, p < 0.001).

#### 3.3.1 Place preference and reversal with reward only

All subjects preferentially visited their rewarded corner as measured by number of visits with nosepoke required to reach the preference criterion, apart from a few exceptional cases (Fig. 6, Reward panel). At Reversal the new preference was reached at the same pace (Chi^2^ = 0.04, df = 1, ns). No significant difference between groups occurred at Place (Chi^2^ = 0.47, df = 2, ns) and Reversal (Chi^2^ = 0.004, df = 2, ns) preference tests. Note that the only consequence of a place error was that the animal had to leave the corner for seeking another one.

#### 3.3.2 Place preference and reversal with incorrect choice punishment

Adding air puff as punishment for nosepoking in the three non-rewarded corners led to a faster acquisition of place preference (Fig. 6, Airpuff vs. Reward at Place preference). Visits required to reach preference criterion with airpuff during Place trial did not differ between groups (Fig. 6, Airpuff panel, Chi^2^ = 1.89, df = 2, ns). However, at Reversal under airpuff augmented learning HIPP subjects required significantly more visits to criterion than CTR and PFC mice (Fig. 6, Airpuff panel, Chi^2^ = 8.95, df = 2, p < 0.02). Accordingly, treatment (Place, Reversal) interacted highly significantly with the Group factor (Chi^2^ = 58.75, df = 2, p < 0.001).

#### 3.3.3 Place preference and reversal during time-Restricted drinking sessions

Preference criterion was reached significantly later in HIPP than in CTR or PFC mice (Fig. 6, Session panel, Chi^2^ = 26.02, df = 2, p < 0.001), independent of treatment (Place, Reversal), as the interaction term was far from significance (Chi^2^ = 0.30, df = 2, ns). Notably, at Reversal, criteria were reached faster than during place preference test in Session trials (Chi^2^ = 7.88, df = 2, p < 0.01), in contrast to reward only trials (see above).

### 3.4 Distinctive effect of hippocampal lesion on spatial working memory tasks

Patrolling paradigms were run twice, without temporal limitations with the first batch of mice, Les1, and during (time-limited) drinking sessions with the second batch, Les2 (see Fig. 2). In the first batch, clockwise conditioning was followed by counter-clockwise conditioning, thereafter clock- and counter-clockwise paradigms were run again but augmented by air puff punishment of nosepokes in incorrect corners (see Fig. 2). However, due to an experimental design error, clockwise with airpuff unintentionally resulted in all mice rewarded exclusively at corner 3, so that this trial with Les1 is excluded from analyses. During counter-clockwise trial with Les2, an intervening drinking trial became necessary due to insufficient drinking by several subjects.

**Fig. 7:**
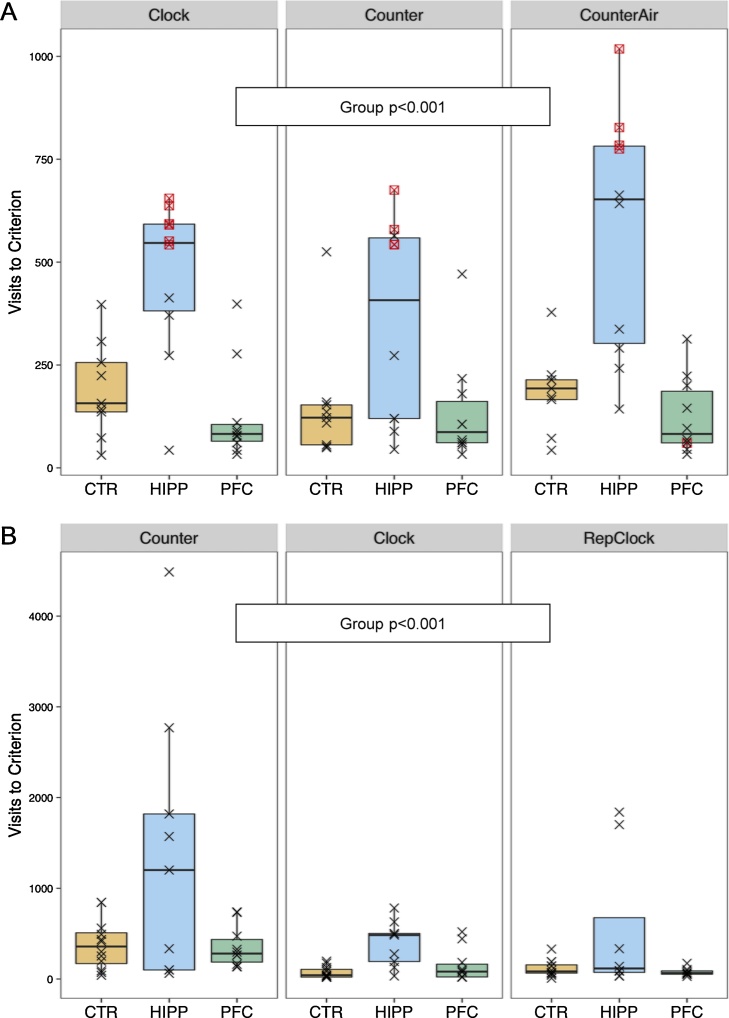
HIPP mice are clearly impaired in patrolling tasks (*Clock* and *Counter*, see Fig. 2) requiring working memory. **A**) Trials to criterion for Les1 patrolling trials with continuous shaping under clockwise, counter-clockwise, and punished counter-clockwise conditioning schemes. **B**) Trials to criterion for Les2 clockwise, counterclockwise, and repeated clockwise conditioning schemes, during restricted drinking sessions. Framed symbols refer to censored observations (i.e., preference criterion was not reached). Panel sequence differs from temporal sequence of schemes (see Fig. 2).

Without temporal limitation, subjects within groups required about the same number of visits to express the preference in clockwise, counter-clockwise, and punished counter-clockwise trials (Fig. 7A). However, HIPP mice performed significantly worse in all trials, with slight but significantly different effect sizes (Group: Chi^2^ = 42.54, df = 2, p < 0.001; Experiment: Chi^2^ = 0.66, df = 2, ns; Interaction: Chi^2^ = 28.16, df = 4, p < 0.001).

With drinking session limitation (Fig. 7B), HIPP mice again took more trials to reach the preference criterion than CTR and PFC mice, but here all groups took longer during counter-clockwise experiment which followed the clockwise run (Fig. 7B), and the difference between groups did not deviate between experiments (Group: Chi^2^ = 25.73, df = 2, p < 0.001; Experiment: Chi^2^ = 24.97, df = 2, ns; interaction: Chi^2^ = 4.55, df = 4, ns). Learning during the final clockwise run did obviously not differ from initial clockwise experiment (Fig. 7B).

### 3.5 Side alternation within corners

**Fig. 8:**
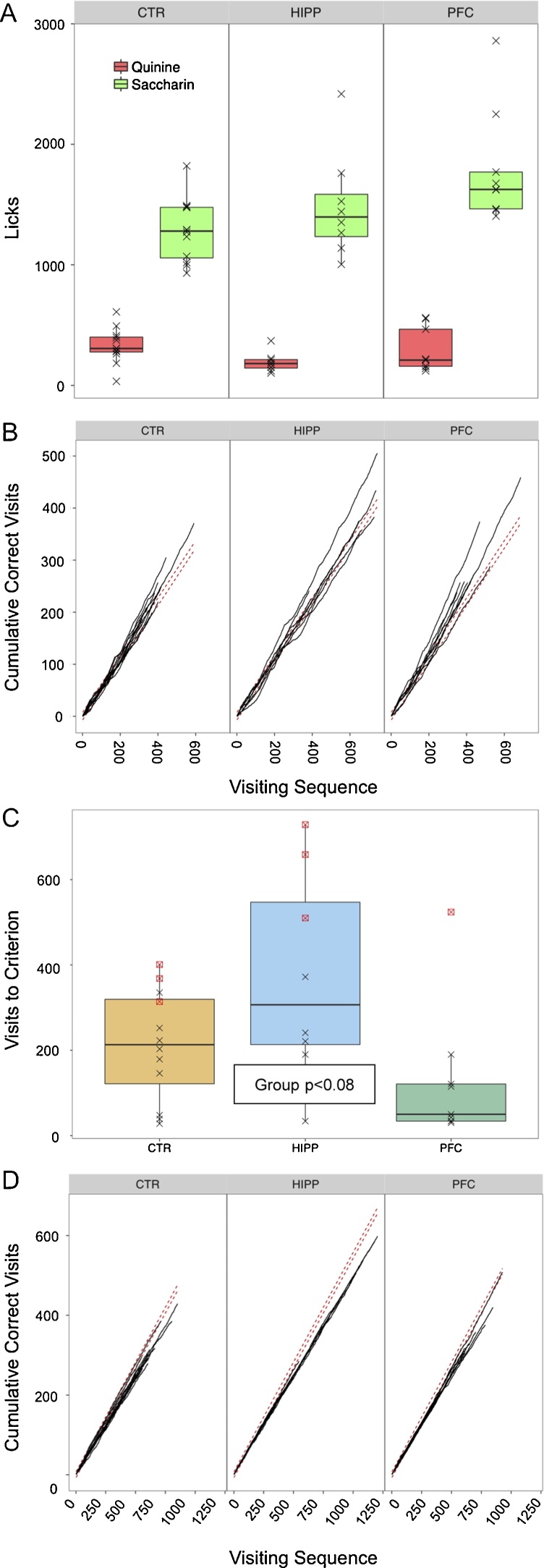
Non-spatial LED stimulus-dependent alternation conditionings failed in all groups, exemplified here for a saccharin preference derived conditioning scheme. **A**) During *SaccQuin* test (see Fig. 2) which offered saccharin solution at one side and quinine solution at the other side of each corner while indicating saccharin side by LED light at corner entry (see Fig. 2 for details of randomization), all subjects exhibited a clear drinking preference for saccharin. **B**) Development of saccharin side choice (placement of first nosepoke of a visit) during *SaccQuin* test. **C**) Visits to criterion of preference for first nosepoke exhibited at saccharin side. Note that most but not all subjects achieved the goal. **D**) Preference for first nosepoke placed at the side lit by green LED after saccharin preference conditioning (*BrightAvoid*_Rep, see Fig. 2). Intention was to condition subjects to nosepoke at the LED lit side (which contained preferred saccharin solution beforehand) at visit entry, when water was given everywhere. However, none of the subjects discriminated accordingly. (For interpretation of the references to colour in this figure legend, the reader is referred to the web version of this article.)

Shaping by LED indication (*LEDside*, *LEDpattern*, *BrightAvoid*, *BrightAvoid_Rep*; see Fig. 2) was intended to identify non-spatial memory impairments, but failed in all cases. Even after most subjects exhibited significant preference for placing the first nosepoke of a visit at the green lit saccharin side during *SaccQuin* preference conditioning (Fig. 8B,C), exhibiting only a slight differentiation between groups (Fig. 8C, Chi^2^ = 5.10, df = 2, p < 0.08), subsequent conditioning on green LED indication failed (Fig. 8D). Hence, effects on non-spatial memory could not be tested. Nonetheless, we present the graphs to indicate the analytical power of the IntelliCage system.

## 4 Discussion

This study shows that a single automated test system containing 8–10 mice per cage with different treatments can identify the hallmarks of large hippocampal lesions such as deficits in spatial reference memory, spatial working memory, impaired handling of conflicting motivational factors or memories, hyperactivity, behavioral stereotypies, and, most notably, a significant disturbance of behavioral timing of spatial schemes. These differences can be observed independent of the order of many behavioral tasks conducted with different cohorts of mice. In contrast to hippocampally lesioned mice, we found mice with lesions of the medial prefrontal cortex exhibiting no cognitive impairments in the paradigms applied, and could only tentatively differentiate them from the other groups by spontaneous activity pattern.

### 4.1 IntelliCage efficiency

The goal of this study was to demonstrate that automated data extraction and analysis of long-term continuous monitoring of sequentially applied conditioning paradigms in the IntelliCage system can evidence detailed behavioral profiles of hippocampal or prefrontal lesion-induced malfunctions. This could obviously facilitate studies on many other topics and mouse models: testing 65 mice in conventional apparatus such as water mazes, T-mazes, activity chambers, operant conditioning boxes, elevated plus mazes and many more would have taken a huge effort requiring a fully equipped and staffed behavioral laboratory. Instead, the only efforts required here after lesioning were the programming of the experimental designs (easily achievable via a graphical interface) that ran automatically in the IntelliCages located in the animal rooms with occasional cage cleaning and data download.

This system, developed by us at the University of Zürich, has not been modified since its commercial introduction in 2006, and is thus representing a truly standardized system avoiding handling-induced biases (see below). Raw data documenting actions of mice and the system are stored permanently in convenient text delimited format, for later extraction and statistical analysis. To allow for automated data analysis at a mouse click, we developed FlowR, a programmable graphical user interface enabling to extract IC data sets by user-defined linking of statistical modules provided by the statistical open-source software R [18]. It allows for efficient extraction and combination of results of various sequentially applied paradigms aimed at testing components of spatial and temporal learning, assessing concomitantly parameters of spontaneous behavior such as activity levels and patterns. The latter point is of importance for analyzing the nature of observed performance deficits in learning tasks. For instance, we compared (data not shown) behavioral scores of spontaneous activity (such as increased number of short visits or repetitivity in corner visits) with learning scores of treated and untreated mice. While the small number of mice precluded sound inferences from structural (causal) models, tentative trends indicated that fruitful inferences might result from larger experiments. The impact of such “non-cognitive’’ behavioral features on learning scores has been shown clearly in the water maze, in which factor analysis showed that variation in acquisition of the task in normal animals is strongly dependent on the degree of wall hugging, and that hippocampal malfunction primarily prolongs strong wall hugging [19], [20], leaving open the question whether this behavior is a matter of cognitive spatial impairment or general perseverance.

A strong point for sequential IntelliCage testing is that mice can be left undisturbed and handled minimally (once a week for changing beddings). There is an increasing amount of evidence that handling by experimenters and subtle differences between them can bias or mask performance in spontaneous behavior and learning tasks [21], [22]. Laboratory-specific factors account specifically for unreliability of unforced tests such as elevated plus mazes and radial mazes [23] while negatively reinforced learning appears more comparable across laboratories [24], [25]. Even subtly different handling of mice can bias test scores [26] and handling times prior to behavioral tests may have aversive effects [27]. Finally, it appears that the odor of male experimenters may stress rats [28], and that even mild novelty stress in mice can entail epigenetic tags potentially biasing later behavioral testing [29]. We do not claim that IntelliCages provide a completely standardized environment, since every cage may contain subtle sources of behavioral variation. However, these did not preclude reliable detection of genetic differences across four laboratories in Europe [30], [31] and Japan and Switzerland [32], [33], in quite remarkable contrast to the notorious inconsistency of inter-lab behavioral scorings with conventional apparatus requiring handling [24]. The advantages of automated testing of hippocampal deficits after lesions have also been emphasized in a study using a T-maze system attached to the home cage, even though the system was testing one mouse at a time only [34].

### 4.2 Lesioning approach

Importantly, lesions were done in fully adult animals (76-141 days) that were then allowed to recover for 51–172 days prior to testing. In addition, the testing period during which they underwent behavioral phenotyping lasted between 154 and 218 days, respectively, in which series of different tasks were applied. Typically, standard post-lesion recovery times in rodents before behavioral testing last between two weeks and one month, with very few studies focusing on temporal development of lesion effects (e.g., [1]). We believe that the use of adult animals after long recovery time abolishes functionally irrelevant and short-lasting alterations, thus showing the true kernel of behavioral deficits attributable to the permanent elimination of the mouse brain regions. For a review of interpreting lesion effects, we refer to [35].

### 4.3 Lack of prefrontal lesion effects

Given the exploding interest in the functions of the prefrontal cortex (about 4400 references dealing with non-human species, PubMed March 2017), the lack of lesion effects in a series of complex learning tasks that should include at least partially the prefrontal cortex appears surprising. However, our results confirm the findings of Deacon et al. [14] who conducted a large number of conventional behavioral tests in C57BL/6 without finding any significant effect, except for reduced anxiety and food-burrowing, itself a strong species-typical behavioral marker for hippocampal malfunction. Likewise, a study comparing prefrontal and hippocampal lesions in mice using chiefly hippocampal tasks, concluded that the prefrontal cortex plays a minor role in the ‘what-where-when’ components of episodic-like memory [36]. Further reasons possibly accounting for this finding are (i) The rather small prefrontal cortex of rats and mice is further partitioned neuroanatomically, possibly associated with different inputs and outputs and functions [37]. Therefore, the lesions might have included different subdivisions whose lesions might result in interacting behavioral effects. (ii) Behaviorally, non-spatial conditioning schemes in IntelliCage based on LED signaling could not be analyzed as there was no apparent learning in control mice (see Fig. 8). (iii) Our study and that of Deacon et al. [14] tested mice after longer than usual postoperative recovery periods, providing enough time for adaptive compensation. One may note that many functional deficits after lesioning parts of the rodent cortex recover within 2–3 weeks (e.g. [38]), one of the reasons that treatments aimed at supporting functional recovery must be conducted within short time windows. Thus, most of the cognitive functions ascribed to the mouse prefrontal cortex regions are not critically dependent on its presence, and can be observed only in the intact brain or within a restricted time window after experimental inactivation.

### 4.4 Hippocampal lesion effects

The canonical discriminant analysis revealed a clear effect on undisturbed spontaneous behavior: HIPP mice, in contrast to both CTR and PFC mice, visited at higher rates for shorter visits while re-entering the previously visited corner closer to random expectation and patterning corner visits less regularly. Hence, HIPP exhibited a more erratic spatial pattern of visiting at higher visiting rates than the other mice. This is congruent with the results from other studies in IntelliCage comparing hippocampal and striatal strain lesions in different mouse strains with behavioral profiles of genetic mouse models [15]. The causal mechanisms underlying these peculiarities remain speculative, but bouts of hyperactivity and loss of behavioral flexibility in spontaneous behavior were commonly reported in older lesion studies, as summarized neatly by O’Keefe and Nadel [8], e.g., p. 255 and Table A14.

Furthermore, adaptation to daily drinking sessions revealed a clear phenotype in HIPP mice which kept on increasing activity prematurely, well before actual drinking sessions started, as shown in Fig. 2b. The effect is noteworthy as it took a clear pattern repeated every day of observation.

#### 4.4.1 Spatial preference tasks with no or little impairment

The most simple form of spatial learning was preference for visiting a given corner where a mouse could have access to water, when doors of the other 3 corners kept closed at visiting (Fig. 6, Reward panel). Such preference was established quickly in all treatment groups after 50–60 visits, except for a few outliers. Reversal learning (water access in a different corner) took a bit longer to learn for all mice but was mastered by HIPP mice equally well. However, one should note that the only consequence of an error (nosepoking in a corner preventing access to the drinking nipples) was the need to move to another corner. Moving more, however, is a weak negative reinforcement for mice that have a strong tendency to move in their home cage, as evidenced amply by their preferences for running wheels whenever available. The ability for rapid spatial preference learning was even more evident when nosepoke errors in the three inappropriate corners were punished by air-puffs: in this condition, all mice learned the task much faster, and there were no more behavioral outliers (Fig. 6, Airpuff/Place panel). One may note that lacking effects of hippocampal lesions on simple spatial learning in rodents (mostly rats) are a common finding [8] (Table A23). Given that all animal species save mammals do not have a hippocampal formation, learning the position of water supply or of the risk for an air-puff within a familiar environment can certainly be done without this brain structure.

Likewise, all mice learned to prefer nosepoking at the rewarded side when every corner contained a side allowing access to water yet punishing nosepokes at the other side (Fig. 4). As a subtle complexity, in two corners the correct side was at left, in the others at right. This set-up in IntelliCage had been used to reveal mild cognitive deficits in mutant mice [39], as it required to differentiate the risks of air-puffs according to the location of the corners. As shown in Fig. 5, the HIPP mice mastered the task also, but showed a trend towards longer acquisition times (p = 0.08), possibly reflecting this cognitive complication.

### 4.5 Spatial tasks with consistent lesion-induced impairments

Clear deficits in HIPP mice emerged when tasks required long-term memory retention, became more ambiguous, included circadian timing of behavioral responses, or patrolling schedules aimed at revealing spatial working memory.

#### 4.5.1 Aversively motivated spatial learning and probe trials

In the place avoidance task shown in Fig. 4a, there was only one among the 4 corners punished by air-puffs after a nosepoke. Punishment consisted either of continuous air-puffs until the mouse had left, or a short puff. HIPP mice were highly significantly impaired in learning this task, regardless of the duration of punishment. Voikar et al. [2] had shown that hippocampals in IntelliCage were more impaired when punishment was continuous, concluding that stimulus salience was possibly more important for response suppression than spatial memory. The data here do not conclusively contradict this conclusion, given the huge variability of the scores among HIPP mice. However, the analysis of the probe trials (cessation of punishment in all corners after 24 h removal from the cage) revealed that HIPP mice re-entered the previously punished corner at chance level almost from start. As evidenced by the smooth plots in Fig. 5, HIPP mice show little evidence of avoidance at probe trial, while CTR and PFC mice appear to keep avoiding the previously punished corner after a short initial phase of indecisiveness that may be attributed to some orientation cue to be re-established. Reduced avoidance may reflect a memory problem in HIPP mice, or lower levels of airpuff aversiveness. The observation that the deficit was smaller after continuous punishment, may be taken as evidence for the latter explanation. However, this seems to be contradicted by the observation, that irrespective of punishment duration, some of HIPP mice persevered in visiting the punished corner at chance or even higher level during the punishing period of the trials. One may note, in this context, that probe trial impairments in the water maze are usually considered as memory deficits.

#### 4.5.2 Aversively motivated reversal learning

When place learning took place with one corner rewarded as well as non-punished, while the other corners responded to nosepoking with airpuffs, HIPP performed well during acquisition yet much worse at reversal (Fig. 6, Airpuff panel). Clearly, initial receiving of an airpuff upon visiting a previously rewarded corner would lead to ambiguousness of spatial cueing that would only be resolved at finding the now rewarded corner. Impaired positional reversal learning after hippocampal lesions or malfunction in mice is regularly observed in the water maze, often more prominent than deficits in probe trial scores [40] when swimming in inappropriate quadrants may be seen as aversive.

Whether the observed deficit reflects response perseverance, ambiguous spatial memory or reduced fear cannot be decided from the data. Since the HIPP mice can learn rapidly a non-punished reversal in the same set-up, a simple memory problem would seem unlikely.

#### 4.5.3 Spatial learning and behavioral timing

Another clear deficit in HIPP mice was found during place as well as reversal learning trials when mice faced the task to obtain water reward only at two given 1 h time periods during conditioning. The task is a circadian-based time-place learning [41] and fits the scheme of episodic-like memory that includes the triple knowledge of what, where and when, which is disturbed in hippocampal lesioned mice [36]. Since the hallmark of (bilateral) destruction of the hippocampus in humans is the loss of episodic memory, there is a wide interest in finding animal models of episodic memory. Interestingly, the peculiar local de-synchronization during *Drink* adaptation (see above) might indicate that circadian triggering is overridden by some more short-term rhythmicity memory hampered by hippocampal lesions. Whether this could be in line with an eliminated intrahippocampal clock sense as postulated by Mulder et al. [41] remains open at present. From a practical point of view, one may note that IntelliCages can be easily programmed to monitor conditioning schemes for analyzing episodic-like memory.

#### 4.5.4 Consistent impairment in spatial working memory tasks

Spatial short-term memory in mice is usually analyzed using T- or Y-mazes for assessing spontaneous alternation, and radial mazes in which mice must patrol baited arms by avoiding repeated visits of the emptied arms. Traditionally, reduced spontaneous alternation [7], [34] and double- and triple-entries in the radial maze [42], [43] are considered a sign of hippocampal malfunction impairing spatial short-term memory. In our study, spatial win-shifting was required during patrolling paradigms where the mice were confronted with the drinking opportunities shifting after each drinking clock- or counterclockwise. In all trials, HIPP mice performed significantly worse than CTR and PFC mice. This may be attributed to hippocampal involvement in spatial disambiguation accounting for win-shift problems after hippocampal lesions as postulated by Bannerman et al. [5].

## 5 Conclusions

1. Automated testing for typical spatial hippocampal tasks in IntelliCage revealed all hallmarks of lesion-induced deficits as reported for water mazes, spontaneous alternation, radial mazes and episodic-like memory in form of circadian-based place-time learning, while not providing evidence for cognitive impairments of mice with lesions of the medial prefrontal cortex. Notably, hippocampally lesioned mice could learn simple place preference and reversal schemes but were impaired when they had to handle concurrent conflicting spatial cues under punishment and temporal restrictions, being likewise impaired in patrolling tasks requiring spatial working memory.

2. In addition, canonical discrimination analysis of spontaneous behavior during adaptation periods revealed clear deviation of activity level and repetitivity as well as regularity of corner visiting in hippocampal mice, but indicated also potential separation of prefrontally lesioned mice which did not reach significance with the small sample at hand. In principle, this would permit evaluations as to the degree non-cognitive behavioral alterations caused by lesions might confound cognitive impairments.

3. Since the lesion-induced changes were observed after long recovery periods and embedded temporally in different conditioning schemes, they must represent the core symptoms of severe hippocampal malfunction that cannot be compensated eventually.

4. To our knowledge, there is no standardized behavioral test system that can provide such a detailed statistical dissection of hippocampal malfunction in the same environment and without interference by experimenters than IntelliCage.
